# *Lm*-LLO-Based Immunotherapies and HPV-Associated Disease

**DOI:** 10.1155/2012/542851

**Published:** 2012-02-02

**Authors:** Anu Wallecha, Chris French, Robert Petit, Reshma Singh, Ashok Amin, John Rothman

**Affiliations:** Advaxis, Inc., 305 College Road East, Princeton, NJ 08540, USA

## Abstract

HPV infection is a direct cause of neoplasia and malignancy. Cellular immunologic activity against cells expressing HPV E6 and E7 is sufficient to eliminate the presence of dysplastic or neoplastic tissue driven by HPV infection. Live attenuated *Listeria monocytogenes-* (*Lm*-) based immunotherapy (ADXS11-001) has been developed for the treatment of HPV-associated diseases. ADXS11-001 secretes an antigen-adjuvant fusion (*Lm*-LLO) protein consisting of a truncated fragment of the *Lm* protein listeriolysin O (LLO) fused to HPV-16 E7. In preclinical models, this construct has been found to stimulate immune responses and affect therapeutic outcome. ADXS11-001 is currently being evaluated in Phase 2 clinical trials for cervical intraepithelial neoplasia, cervical cancer, and HPV-positive head and neck cancer. The use of a live attenuated bacterium is a more complex and complete method of cancer immunotherapy, as over millennia *Lm* has evolved to infect humans and humans have evolved to prevent and reject this infection over millennia. This evolution has resulted in profound pathogen-associated immune mechanisms which are genetically conserved, highly efficacious, resistant to tolerance, and can be uniquely invoked using this novel platform technology.

## 1. Introduction

It has been estimated that HPV infection accounts for approximately 5 percent of all cancers worldwide [1]. Persistent HPV infections are now recognized as the cause of essentially all cervical cancers. In 2010, it was estimated that about 12,000 women in the United States would be diagnosed with this type of cancer and more than 4,000 would die from it. Cervical cancer is diagnosed in nearly half a million women each year worldwide, claiming a quarter of a million lives annually. HPV infection also causes some cancers of the anus, vulva, vagina, and penis [1]. Sexually transmitted HPV infections are very common and have peak prevalence between the ages of 18 and 30. Most of these infections resolve spontaneously, but, in 10–20% of women, these infections remain persistent and are at risk of progression to Grade 2/3 cervical intraepithelial neoplasm (CIN) and eventually to invasive cancer of the cervix (ICC). CINs are genetically unstable lesions with a 30–40% risk of progression to ICC. If left untreated, they form a spectrum of increasing cytological atypia, ranging from low-grade CIN 1 to high-grade CIN 3; the latter are caused almost exclusively by high-risk HPVs, namely, HPV 16 and 18.

Oral HPV infection causes some cancers of the oropharynx (the middle part of the throat, including the soft palate, the base of the tongue, and the tonsils) [1–5]. HPV is associated with 20–50% of oral squamous cell carcinomas [6], and the incidence is dramatically increasing. HPV has also been implicated as having a role in certain colorectal cancers and lung cancers although the association is somewhat controversial.

## 2. HPV as a Target for Immunotherapy

HPV is a double-stranded, circular DNA virus devoid of an envelope. Depending upon the strain, its genome contains either six or seven early proteins (E1, E2, E4, E5, E6, E7, and E8) and 2 structural proteins which appear later (L1 and L2). Infection with the virus occurs in replicating, differentiating basal epithelial cells. As keratinocytes differentiate and mature, the expression of viral genes results in viral protein production until terminally differentiated surface cells express the late proteins, the viral capsid is assembled, and the virus is shed. HPV-induced cancer can occur when viral DNA integrates into the genome of the host, typically with the deletion of the genes E2, E4, E5, L1, and L2. The loss of the viral E2 gene, which is a transcriptional inhibitor, leads to the upregulation of two oncoproteins from genes E6 and E7. The viral oncoprotein E6 complexes with the tumor inhibitor gene *p53* and the oncoprotein E7 complexes with the tumor suppressor protein retinoblastoma (pRb) [10], disrupting cell cycle regulation and leading to genomic instability and subsequent neoplasia [11].

HPV-associated neoplasia is one of the most clear-cut situations in medicine where infection with an exogenous agent (a virus) is a direct cause of neoplasia and malignancy. Cellular immunologic activity against cells expressing HPV E6 and E7 is sufficient to eliminate the presence of dysplastic or neoplastic tissue driven by HPV infection. The variable but significant rate of spontaneous remission is felt to be due to immunologic recognition of the HPV proteins expressed in transformed cells and higher numbers of CD8^+^ cells and a higher ratio of CD8^+^/FOXp3 cells infiltrating the dysplastic tissue [4, 12–14]. An immunologic stimulus may be required to overcome tolerance that has developed to the HPV-transformed dysplastic cells.

## 3. Use of *Lm*-LLO Immunotherapy for HPV-Associated Disease

A therapeutic change in the ratio of CD8^+^ TIL to Tregs has been observed as a result of the administration of *Lm*-LLO immunotherapies in a variety of models [15–18]. *Lm*-LLO-E7 (ADXS11-001) has been found in a variety of preclinical models to stimulate immune responses and affect therapeutic outcomes and is currently in clinical trials.

Just such a live attenuated *Listeria monocytogenes*- (*Lm-*) based immunotherapy (ADXS11-001) has been developed for the treatment of HPV-associated diseases by Advaxis, Inc. ADXS11-001 secretes an antigen-adjuvant fusion (*Lm*-LLO) protein consisting of a truncated fragment of the *Lm* protein listeriolysin O (LLO) fused to HPV16-E7. A Phase 1 study has been completed with ADXS11-001 [19] and 4 Phase 2 clinical trials are active or about to be initiated.

*Lm*-LLO immunotherapies have multiple simultaneous mechanisms of action that can summate in a therapeutic response [20]. *Lm* stimulates innate immunity and infects APC where it naturally cross-presents to stimulate both arms of the adaptive immune system resulting in activated CD4^+^ and CD8^+^. These agents reduce intratumoral Tregs and MDSC, but not those in spleen or lymph nodes. They can stimulate the maturation of immature immune cells to terminally differentiated effector cells and shift the kinetics of bone marrow to produce increased numbers of myeloid cells. Effects have been observed in vascular endothelial cells to facilitate chemotaxis and the extravasation of activated immune cells. *Lm* is an entirely cellular immune stimulating agent, and antibody formation of the type that can inactivate viruses does not occur with *Lm*. Interestingly, consolidated immune memory responses to *Lm* antigens have been observed to occur rapidly, with correlates of immune memory to *Lm* occurring as early as 5 hours after exposure [21].

The use of a live attenuated bacterium is a different way to approach cancer immunotherapy than those based upon synthetic chemistry or antibody-based agents. It is more complex, as *Lm* has evolved to infect humans and humans have evolved to prevent and reject this infection over millennia. This evolution has resulted in profound pathogen-associated immune mechanisms which are genetically conserved, highly efficacious, and can be uniquely invoked using this novel platform technology.

## 4. *Listeria monocytogenes (Lm)*: A Potent Vector for Immunotherapy for Neoplastic and Infectious Disease

Previous studies have shown that bioengineered *Lm* is a potent vector not only for immunotherapy of cancer but also for infectious diseases [20, 22]. This makes HPV infections and consequently HPV-associated cancers a prime target for therapy. With *Lm-*LLO-based immunotherapy, it is possible to (a) eradicate tumors induced by HPV and (b) prevent reoccurrence of the tumor. Advaxis in collaboration with Yvonne Paterson's Lab (University of Pennsylvania School of Medicine) has developed various vectors expressing the tumor-specific antigens (TSAs) that target HPV-induced cancer as described in Table 1. Preclinical studies using different plasmid backbones for delivering E7 show similar antitumor therapeutic efficacy in all the vectors described in Table 1. The ADXS11-001 (*Lm*-LLO-E7) was selected for human studies as it was extensively studied and tested in preclinical settings. Furthermore, ADXS11-001 pathogenicity was attenuated by 10^4^ to 10^5^ logs, compared to the wild-type *Lm* parent strain 10403S, thus increasing its safety for clinical use.

Various methods of bioengineering allow *Lm* to express TSA on the plasmid or in the genome via chromosomal insertion [23, 24]. At Advaxis, two complementation mechanisms have been designed for the *in vivo* retention of plasmids in attenuated bacterial strains. One strain is a *prfA *deletion mutant which is avirulent due to the absence of the master virulence regulating protein P*rfA*, rendering it unable to escape the phagolysosome, but the intracellular growth ability is restored through the complementation of P*rfA* on a plasmid. This complementation ensures *in vivo* retention of the plasmid, but for *in vitro* manipulation antibiotic resistance markers such as chloramphenicol resistance gene were used [7, 8]. Another backbone is a mutant strain defective for D-alanine synthesis, which is essential for bacterial cell wall synthesis. Survival of *Lm* strain deficient in *dal* and *dat* genes depends upon the plasmid-based complementation of the *dal* gene. To eliminate the possibility of recombination of *dal* gene present in the plasmid and the *Lm *genome, *Bacillus subtillis dal* gene was used for complementation of *in vivo* and *in vitro *growth. This complementation not only creates an antibiotic-marker-free plasmid delivery system but also attenuates the vector by 0.5 to 1 log [9]. ADXS11-001 with the *prfA* deletion was found to be cleared by SCID mice using innate immunity alone [20], and the clearance kinetics of the *dal dat* demonstrated clearance within 72 hours. A highly attenuated Lm* dal^−^ dat^−^ actA^−^* (*Lm*ddA) backbone was created at Advaxis [24], which is cleared rapidly *in vivo* and contains an antibiotic-marker-free plasmid for expression of TSA, which is strong candidate for immunotherapy in the clinic. Similar *in vivo* clearance of *Lm*ddA strain in both normal and interferon-gamma knockout mice demonstrates that this strain is highly attenuated and safe for clinical use.

## 5. LLO: An Adjuvant for Immunotherapy

Listeriolysin O is a hemolytic, thiol-activated, cholesterol-dependent pore-forming protein which is essential for intracellular escape of *Lm* from the phagolysosome [25]. Recent advances in immunology have resulted in a number of potential adjuvant candidates that are able to modulate the immune response in a more controlled and specific manner [26]. These adjuvants modulate and target specific immune components, such as activation of different cells, receptors, or signaling pathways. [26]. Advaxis studies show that nonhemolytic LLO also harbors unique properties of an adjuvant: (a) augments the effects of “non-self-foreign” antigens as do classical adjuvants, (b) breaks tolerance of “self-/tumor-associated antigens,” (c) specifically activates or augments functions involved in antitumor activity, (d) regulates complex soluble mediators and their receptors to optimize the antitumor activity, and (e) modulates signals to activate different arms of the immune systems for antitumor activity. Gunn et al. engineered an LLO molecule truncated at the C-terminal of the protein, which rendered the LLO nonhemolytic [20]. Neeson et al. [27] independently reported that LLO has adjuvant properties when used in the form of a recombinant protein vaccine. Fusion of LLO to tumor antigens delivered by other vaccine modalities, such as viral vectors [28] and DNA vaccines [29], also enhances their therapeutic efficacy. These properties of recombinant LLO positions it as an attractive adjuvant not only for breaking local and peripheral immunological tolerance of tumors and associated antigens but also for mounting an antigen-specific and antigen-coordinated anticancer immune response as described followingly in mouse models for HPV-related cancer.

## 6. Intracellular Events and Antigen Presentation of *Lm-*LLO-Ag (HPV) Fusion Protein

**Figure 1:**
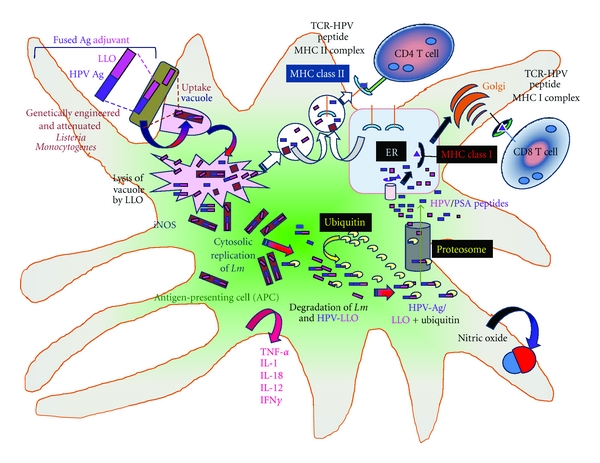
Schematic presentation of LLO-Ag (HPV) fusion protein processing and presentation in antigen-presenting cell (APC) by *Listeria monocytogenes*. Advaxis in collaboration with Paterson et al. has developed human papilloma virus (HPV) and listeriolysin (LLO) fusion proteins in *Lm *for immunotherapy [17, 22–24]. Upon injection *in vivo*, these *Lm *are sequestered and engulfed by antigen-presenting cells (APCs) such as dendritic cells [23, 24, 31]. The bacteria are engulfed by vacuoles where most of the *Listeria* are killed [18, 32]. The bacterium while processing the tumor-associated antigen (HPV) and listeriolysin O (LLO) stimulates both arms of the adaptive immune system [20, 34]. Part of the antigen from the vacuole is processed via the MHC class II molecules which generate CD4^+^ T cells. Five to ten percent of these *Lm *escape into the cytosol with the assistance of the LLO where *Listeria* can undergo replication. The cytosolic HPV-LLO fusion protein behaves as endogenous antigens. The HPV-LLO fusion protein undergoes ubiquitination, and it is processed via the proteasome [20]. The resulting peptides are presented via the MHC class I molecules to generate CD8^+^ T cells [34]. These cells generate strong cell-mediated immune responses. *Lm *also evokes a strong innate immune response which leads to generation of numerous mediators such as nitric oxide which is involved in killing of the bacteria in the vacuoles and cytokines (such as TNF-*β*, IL-1, IL-18, IL-12, and IFN*γ*) which impart several types of bystander effects [20, 33, 35–37].

As shown in Figure 1, attenuated *Lm *carrying the HPV antigen fused to LLO can be phagocytized by antigen-presenting cells, macrophages, and other cells [20, 30]. The attenuated bacterial cells are taken up into the endosome where they evoke a conserved pathogenic assault [31] and redirect the tumor antigen [23]. The PEST-like sequence of LLO is important as it has been shown to increase antitumor efficacy of *Lm*-based vectors expressing the fusion protein LLO-PEST-E7 in HPV-16 immortalized tumors [8]. This process stimulates cell-mediated immune response generating CD4^+^ cells and CD8^+^ T cells [20]. The fusion of antigens to LLO facilitates the secretion of the antigen [32] and increases antigen presentation [8] with a profound influence on CD8^+^ T-cell activation [20, 33].

## 7. *In Vivo* Response to *Lm*-LLO-Ag (HPV) Fusion Protein and Cellular Events in the Tumor Environment

**Figure 2:**
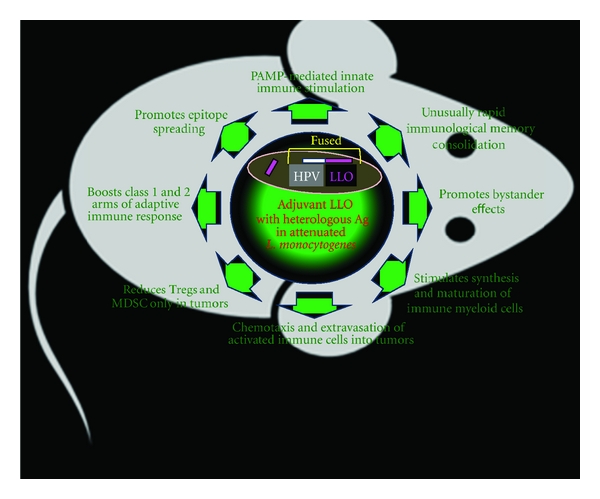
Hypothetical representation of *in vivo* effects of *Lm-*LLO-based immunotherapy. *Lm*-LLO-based immunotherapy evokes a cascade of events *in vivo* which involves multiple cell types that may (a) regress existing tumors and (b) block tumor reoccurrence. The physiological events associated with these potent therapeutic and prophylactic events include the following: (1) unusually rapid immunological memory consolidation is generated with five-hour post-*Listeria*-based immunotherapy [41, 42]; (2) promotes bystander effects via activation of cytokines, chemokines, and/or their receptors regulate functions such as leukocytosis, memory, and listeriosis [20]; (3) stimulates synthesis and maturation of immune myeloid cells by stimulating formation of myeloid cells and maturation of dendritic cells [39, 43, 44]; (4) guides heterologous Ag (HPV) processing to generate antigen-specific CD4^+^ and CD8^+^ cells, via MHC class II and I pathways, respectively [34, 43]; (5) reduces Tregs and MDSC only in tumors and diminishes the tumor's resistance to immune attack by antigen-specific cells [4, 14, 20, 45]; (6) boosts class 1 and 2 arms of adaptive immune response which generates strong cell-based antitumor immunity [9, 24, 30]; (7) chemotaxis and extravasation of activated immune cells is part of an innate immune response, involving the recruitment of nonspecific leukocytes into tumors [34, 46, 47]; (8) PAMP-mediated innate immune stimulation facilitates processing of live *Listeria* which evokes the essential activity of inflammasomes and innate immunity [48].

An *in vivo* response to *Lm-*LLO-Ag (HPV) fusion protein induces several immune functions which are well coordinated to break the local and peripheral tolerance to tumor-specific antigens and to initiate a chain of antitumor activities utilizing various soluble mediators and cells as shown in Figure 2. LLO is a potent inducer of inflammatory cytokines, such as IL-6, IL-8, IL-12, IL-18, TNF-*α*, IFN-*γ*, and GM-CSF; nitric oxide, chemokines, as well as costimulatory molecules that are important for innate and adaptive immune responses [20, 35–37]. One example of the high Th-1 cytokine-inducing activity of LLO is that protective immunity to *Lm *can be induced with killed or avirulent *Lm *when administered together with LLO, but not in the absence of LLO [38]. Cytokines induced in macrophages in the presence of LLO [39] in turn activate NK cells to release IFN-*γ* [40].

## 8. Generation of Tumor-Antigen-Specific Cytotoxic T Cells and Regression of HPV-Associated Tumors

**Figure 3:**
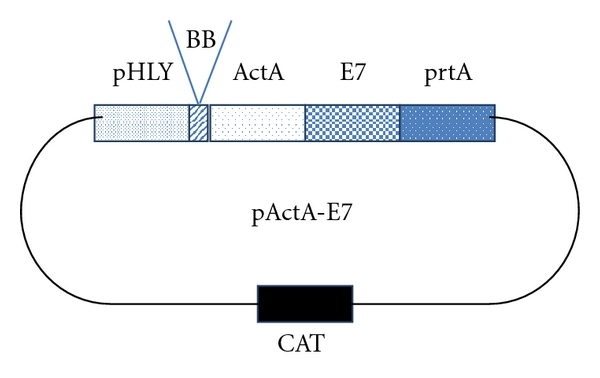
A schematic representation of the plasmid pActA-E7. The recombinant plasmid was used to transform the* Lm *strain XFL-7 to create *Lm*-ActA-E7. The vector includes a promoter (*pHly*) and signal sequence (ss) from the *hly *gene, the *actA* gene, the human papillomavirus 16 E7 gene, and the transcription factor *prfA*. XFL-7 is a *prfA*-deleted strain of *Lm.* Thus, only bacteria that retain the plasmid will replicate *in vivo*. Adopted and modified from [49].

Preclinical studies using a genetically engineered attenuated strain of *Lm *expressing HPV-16 E7 demonstrated therapeutic activity against E7-expressing tumors in animal models [7]. Two *Lm*-LLO-based immunotherapy vectors, one of which expresses the antigen HPV-16 E7 alone and one which expresses E7 fused to a truncated form of LLO, showed regression of the E7-expressing tumor, TC-1, established in syngeneic C57BL/6 mice [7]. A lung epithelial cell line (TC-1) immortalized with HPV-16 E6 and E7 and transformed with the c-ras oncogene was used in these studies. Paterson et al. have recently utilized a new recombinant strain of *L* that uses a multicopy episomal expression system (*Lm*-ActA-E7) to secrete the HPV protein E7 fused to the *Lm *protein ActA as shown in Figure 3.

**Figure 4:**
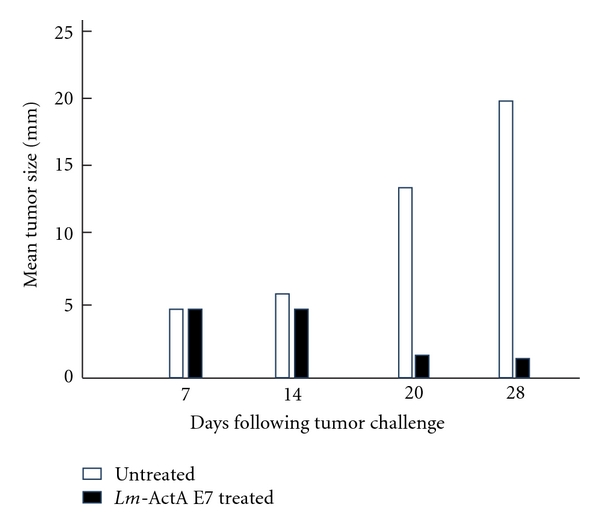
*Lm*-ActA-E7 causes regression of established TC1 tumors. C57BL/6 mice received 2 × 10^5^ TC1 cells subcutaneously on the left flank. Tumors grew to 5 mm after 7 days. The mice were then treated with 0.1 median lethal dose of *Lm*-ActA-E7 or *Lm*-LLO-NP (data not shown) as a negative control on day 7, and a booster dose was given on day 14. The third and final group was left untreated. The average tumor diameter was measured with calipers and is shown for each mouse. The difference in tumor sizes between the *Lm*-ActA-E7 group and either control group at days 20 and 28 is statistically significant (*P* ≤ .005 and *P* ≤ .001, resp.). Depicted is 1 experiment representative of 4. The figure and legend were adopted and modified from [49].

The *Lm*-ActA-E7-based immunotherapy (but not *Lm*-ActA-NP treated—used as nonspecific—controls) or untreated controls caused 75% regression of the HPV-positive tumors on day 20 when compared to the established tumor on day 7. However, more than 90% regression of tumors was observed when *Lm*-ActA-E7-induced tumor reduction as compared to controls on day 28 (Figure 4).

**Figure 5:**
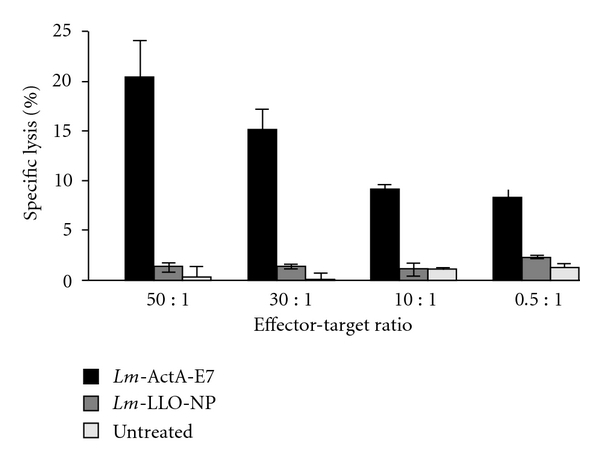
*Lm*-ActA-E7 induces E7-specific cytotoxic T-lymphocyte (CTL) activity. C57BL/6 mice were immunized with 0.1 median lethal dose of *Lm*-ActA-E7 or Lm-LLO-NP. A separate group of mice was left untreated. A booster immunization was administered 7 days later. Splenocytes were harvested 7 days after the booster and established in primary culture with irradiated TC1 cells for 7 days. Following the primary culture, CTL activity was assayed via chromium 51 (^51^Cr) release from EL4-E7 cells. The CTL activity was significantly higher in those mice that were vaccinated with *Lm*-ActA-E7 than in controls (*P* ≤ 0.05). Results are expressed as the mean of triplicate cultures. These results are representative of 3 experiments. The figure and legend are adopted from [49].

Sewell et al. showed that antitumor activity of *Lm*-LLO-based immunotherapy against E7 could also be seen in solid tumors implanted in transgenic mice [8, 49]. This model system also revealed the enhanced antitumor efficacy of *Lm-LLO*-based vectors expressing the fusion protein LLO-PEST-E7 in HPV-16 immortalized tumors in syngeneic mice. It should be noted that this immunotherapy has the potential not only to cause tumor regression but also to prevent the recurrence of tumors. A cytotoxic T-lymphocyte assay revealed that administration of *Lm*-LLO-based vector caused the generation of cytotoxic T cells specific for E7 (Figure 5).

## 9. Ability of *Lm*-LLO-E7 to Induce CD8^+^ T-Cell Memory and Regression of Established Tumors after Antibiotic Administration

**Figure 6:**
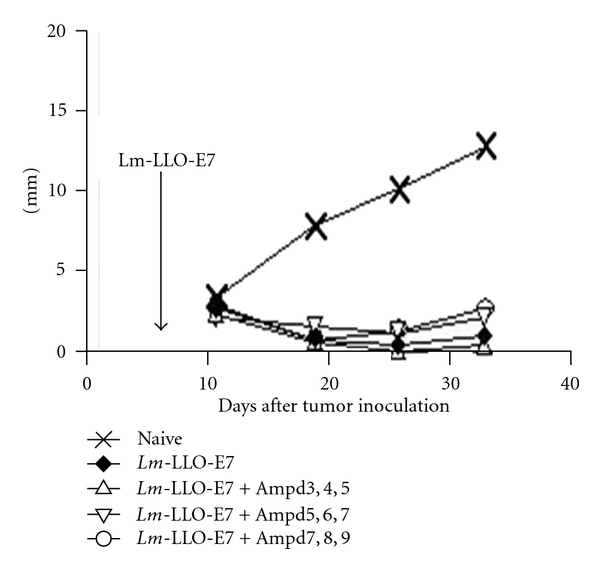
Effect of ampicillin treatment on therapy of TC1 by ADXS11-001. C57BL/6 mice were injected with 1 × 10^5^ TC1 tumor cells. Seven days later, the mice were treated with 0.1 × LD_50_ of ADXS11-001. Beginning 3, 5, or 7 days after ADXS11-001 treatment, some of the mice received daily injections of 10 mg of Ampicillin, delivered for three consecutive days; the mice were then maintained on drinking water supplemented with Ampicillin at a concentration of 0.5 mg/mL. The data is adopted from [17].

It should be noted that although *Lm-*LLO-based immunotherapy required a live attenuated bacteria as a carrier of the fusion antigen, the bacteria may be killed shortly after administration by antibiotic treatment and the immunotherapy will continue to demonstrate antitumor activity [20]. Experiments in mice by Bajénoff et al. showed that *Lm*-specific and *Lm*-nonspecific memory CD8^+^ T cells could be observed within 6 hours of infection and with *Lm* burden [41]. The *Lm*-specific and *Lm*-nonspecific memory CD8^+^ T cells were localized in red pulp of the spleen which formed clusters around *Lm*-infected cells. Memory CD8^+^ T cells produced inflammatory cytokines such as IFN-*γ* and CCL3 nearby infected myeloid cells which are known to be crucial for *Lm* killing. Corbin and Harty [42] reported that *Lm*-infected mice treated with antibiotics at 24 hours postinfection showed a robust increase in antigen-specific CD4^+^ and CD8^+^ T cells similar to the response in controls that did not receive the antibiotics. Furthermore, antibiotic treatment did not alter secondary antigen-specific T-cell expansion or protection with or without the antibiotics [42]. These experiments demonstrate that development of early CD4^+^ and CD8^+^ T cells show functional memory, independent of prolonged infection or antigen display on day 28.  Figure 6 shows that administration of antibiotics on day 3 posttreatment with ADXS11-001 has no effect on efficacy as more than 90% tumor regression occurred in mice.

## 10. Intracellular Milieu in Tumors

The presence of a complex immune suppressive network in the tumor microenvironment includes, but is not limited to, (a) Tregs, (b) myeloid-derived suppressor cells (MDSCs) along with their mediators (i.e., IL-10, TGF-*β*, GM-CSF, PGE_2_, B7-H1, PD-1, and Arginase I), (c) functionally impaired immune cells, and (d) tumor-associated macrophages (TAMs) and their mediators such as nitric oxide which effectively halts the antitumor immunity [45]. The intracellular milieu is a challenging aspect for any immunotherapy including *Lm*-LLO-based immunotherapy. Figure 2 summarizes some of the *in vivo* events manifested by *Lm*-LLO-based immunotherapy which have the ability to neutralize and/or reverse cell functions and mediator release involved in tumor immunity. Much of these events are also induced in the animal model of E7-induced tumors during *Lm*-LLO-HPV-induced immunotherapy. For example, studies by Advaxis and Paterson Lab showed a correlation between CD8^+^ T-cell induction, tumor homing, and the antitumor efficacy of the *Lm-LLO*-based immunotherapy [20].

The effect on different T-cell populations in tumor microenvironment after treatment with *Lm*-E7 and *Lm*-LLO-E7 in mice harboring TC1 tumors is shown in Table 2. There was an increase in TILs and a decrease in CD25^+^CD4^+^FoxP3^+^ Tregs in tumors of mice immunized with *Lm*-LLO-E7 suggesting that LLO-E7 fusion not only increases T-cell infiltration but also reduces suppressive cells intratumorally. In order to determine if similar effect on Tregs was observed in the periphery, the distribution of these cells was monitored in the spleen. As shown in Table 3 treatment with *Lm*-LLO-E7 vaccine causes a preferential decrease in the Tregs intratumorally and has no effect on the periphery such as spleen. These studies show that *Lm*-LLO-based immunotherapies cause specific reduction of Tregs within the tumor to stimulate antitumor immunity.

The fusion of antigens to LLO also appears to facilitate the secretion of the antigen [7, 32] and increased antigen presentation with a profound influence on the CD8^+^ T-cell activation [50]. *Lm*-LLO-Ag reduces the percentage of immune-suppressive Tregs infiltrating the tumor and helps to stimulate the maturation of DCs and other myeloid cells [44]. Singh et al. have shown a decrease in MDSC to play a critical role in tumor regression with *Lm-*LLO-based immunotherapy in mouse cancer models (unpublished data. Advaxis, Inc.). Previous studies have reported accumulation of *Lm* within the tumor during immunotherapy [20]. *Lm-*based vaccines have been reported to infect the primary tumor and metastases tumor *in vivo *[51]. Kim et al. [51] suggested that *Lm* vaccines could kill tumors (a) by directly infecting the tumor and increasing the levels of ROS and (b) by directing CTL responses against cells expressing specific antigens.

Preclinical studies demonstrate that *Lm*-LLO-based immunotherapy encompasses a coordinated and comprehensive cellular reaction towards tumor destruction in E7-induced tumors in mouse models. These preclinical data show that *Lm*-LLO-based immunotherapy is pleotropic in nature and has many of the traits required for overcoming the central and peripheral immunological tolerance that is exerted in the tumor microenvironment described above. Furthermore, *Lm*-LLO-based immunotherapy is antigen, tissue specific, and unlike chemotherapy, once the tumor is eradicated, it persistently blocks its reoccurrence in mouse models of cancer due to the development of immunological memory. These experiments also demonstrated the efficacy of *Lm-*LLO-based immunotherapy to a tumor that is induced by a viral oncogene.

## 11. Clinical Development Plan for ADXS11-001

The most likely diseases to evaluate the safety and efficacy of ADXS11-001 are cervical intraepithelial neoplasia (CIN) and cervical cancer, HPV-positive head and neck cancer, and perhaps other HPV-associated diseases like vulvar intraepithelial neoplasia (VIN), and even lung and colorectal cancer where an HPV link can be identified. To date, a Phase 1 study has been completed, two Phase 2 trials are ongoing, and 2 additional Phase 2 trials currently await institutional approval to begin.

### 11.1. Phase 1 Study

A Phase 1 trial of ADXS11-001 was conducted in 15 patients with previously treated metastatic, refractory, or recurrent cervical cancer who had failed previous cytotoxic therapy [19] and in a population where no therapeutic regimen had been shown to extend survival. ADXS11-001 was administered by intravenous infusion at three (3) dose levels (1 × 10^9^ CFU, 3.3 × 10^9^ CFU, and 1 × 10^10^ CFU) using a dose escalation design across cohorts with each patient in a cohort receiving only two administrations of the same dose. The infusion was administered to each study participant over 30 minutes and occurred once every 21 days for a total of two treatments on days 1 and 22, respectively. Overall, 15 (100%) of patients experienced cytokine-mediated adverse events (AEs). The most commonly reported AEs were pyrexia, chills, anemia, headache, vomiting, nausea, tachycardia, and musculoskeletal pain. Drug-related AEs were mild to moderate, transient in nature, and consisted of “flu-like” symptoms such as pyrexia, vomiting, chills, headache, tachycardia, and nausea and which responded to standard nonprescription agents. Infusion of 1 × 10^10^ or more CFU without premedication resulted in a dose limiting toxicity (DLT) of Grade 2 diastolic hypotension occurring within hours after the ADXS11-001 infusion that required therapeutic intervention. In all patients, the hypotension was successfully controlled with IV fluids and supporting medication. Similar DLT have been observed at 1 × 10^10^ for other live *Lm*-based vectors in trials conducted by other sponsors [19]; therefore, doses of 1 × 10^9^ CFU or less were selected for subsequent clinical evaluation.

Historically, the median survival of these patients is approximately 6 months with a one-year survival of 5% (unpublished data (GOG 127 series Phase 2 studies)). In the Phase 1 study of ADXS11-001, 4 of 13 evaluable patients experienced a reduction of their tumor burden; median survival was 347 days, and one-year survival was 53%. 11/15 patients (73%) had a performance status ECOG 2–4. The clinical benefit of increased survival and tumor shrinkage observed in this advanced malignancy setting merited further investigation.

### 11.2. Phase 2 Studies

Most immunotherapies seem to work best in earlier stages of disease where the tumor burden is lower and there has been less prior therapy. In the case of HPV-associated cervical cancer, there is a clear and slowly progressing maturation of dysplasia toward cervical cancer known as CIN. Frequent Pap smears and colposcopic examination can identify subjects with CIN. The standard of care for high-grade CIN (CIN 2/3) is a surgical resection of the dysplastic tissue in the cervix. While this is typically an outpatient procedure, it can compromise future fertility of the woman and recurrence can occur. There is also a significant spontaneous remission rate in women with CIN which is inversely proportional to the grade of their CIN. An agent that can induce immunologic remission of high-grade cervical dysplasia could eliminate the risks associated with surgery and provide immunologic memory that could in theory protect against recurrence.

*Lm*-LLO-E7-07 is a randomized, single blind, placebo-controlled, dose escalation Phase 2 trial being conducted in the US in 120 women with CIN 2/3. The initial 40 subject cohort has been completed with 31 subjects receiving 80 doses. Each subject received 3 doses each of dose 5 × 10^7^ CFU or placebo (3 : 1 randomization). Enrollment of the second cohort is ongoing.

*Lm*-LLO-E7-015 is a randomized Phase 2 trial being conducted in India in women with progressive cervical cancer who have failed cytotoxic therapy. Patients are randomized to 3 doses of ADXS-011 at 1 × 10^9^ CFU or 4 doses of 1 × 10^9^ CFU with cisplatin chemotherapy between doses 1 and 2. As of 8/1/11, 54 patients have received 117 doses.

In both studies, Naprosyn and oral promethazine are given as premedications to ameliorate potential side effects, and a course of ampicillin is given 3 days after infusion as a precautionary measure. From this clinical experience, a clear pattern of treatment-related adverse events has emerged consisting of fever, chills, nausea, and vomiting which are consistent with the release of immunologic cytokines commonly associated with immune activation. Between 15 and 23% of the doses administered have been associated with a drug-related adverse event; typically a transient Grade 1 or 2 (mild-moderate) flu-like symptom, which appears within a few hours to 3 days after infusion. Symptoms either self-resolve or respond quickly to symptomatic treatment. Thus far, there have been no serious adverse events associated with ADXS11-001 in 171 doses, no evidence of listeriosis, no persistent symptoms, no delayed symptoms, and no evidence of cumulative toxicity in subsequent doses.

A GOG Phase 2 trial in the US in patients with recurrent/refractory cervical cancer and a Phase 1/2 safety and efficacy of ADXS11-001 in HPV-positive oropharyngeal head and neck cancer were funded by Cancer Research UK (CRUK).

## 12. Conclusion

ADXS-11-001 immunotherapy can be safely administered to healthy young subjects as well as patients with advanced cancer and presents a predictable and manageable safety profile. This agent has the capability of inducing the type of immunologic response that has been observed in cases of spontaneous remission and responding HPV-transformed lesions. ADXS-11-001 can generate a Th-1 type immunologic response generating CD8^+^ T cells that target HPV-E7-transformed cells while simultaneously suppressing the Treg- and MDSC-driven immunologic tolerance within the lesions, increasing the CD8/Treg(FOX P3+) ratio, and causing clinical remission. Clinical trials are ongoing to evaluate the activity of this agent across the spectrum of diseases caused by HPV transformation from cervical intraepithelial neoplasia (CIN 2/3) through locally advanced cervical cancer to advanced recurrent cervical cancer. Other HPV-associated malignancies are also being investigated or are of interest including HPV-positive head and neck cancer and types of lung and colorectal cancer where an HPV link can be identified.

## Figures and Tables

**Table 1:** Modification of *Lm-*LLO-based immunotherapy for HPV-associated cervical cancer.

Vaccine name	Design	Strain modification	Antigen	Ref.
ADXS11-001 (*Lm*-LLO-E7)	Plasmid	*prfA* ^−^	E7, HPV-16	[7]
*Lm*-PEST-E7	Plasmid	*prfA* ^−^	E7, HPV-16	[8]
*Lm*-ActA-E7	Plasmid	*prfA* ^−^	E7, HPV-16	[8]
*Lm*-dd-TV	Plasmid	*dal* ^−^ *dat* ^−^	E7, HPV-16	[9]

**Table 2:** *Lm-*LLO-based immunotherapy increases CD8^+^ T cells (TILs) and decreases CD25^+^CD4^+^FoxP3^+^ Tregs in tumor. Comparison of CD8^+^ T cells (TILs) and CD25^+^CD4^+^FoxP3^+^ Tregs in TC1 mouse tumor after treatment with *Lm*-E7 or *Lm*-LLO-E7. The data has been adopted and modified from Shahabi et al. [17].

Immunotherapy group	E7/Db tetramer positive activated CD8^+^ T cells in tumors (TILs)	CD25^+^CD4^+^FoxP3^+^ Tregs in the tumor	CD8^+^ TIL : Tregs ratio
*Lm*-E7	9.4%	11.8%	0.80
*Lm*-LLO-E7	36.8%	1.7%	21.65

**Table 3:** Percent of intratumoral Tregs in a TC1 model following treatment with *Lm*-E7 and *Lm*-LLO-E7 in mouse model of cervical cancer [20].

Percent intratumoral Tregs by vector type			
*Lm*-E7		*Lm*-LLO-E7	
Spleen	Tumor	Spleen	Tumor
6.4	12.1	4.5	2.3
7.0	12.2	3.9	2.0
6.9	14.9	4.5	1.1
6.5	8.9	3.9	1.3
